# A Survey on Underwater Acoustic Sensor Network Routing Protocols

**DOI:** 10.3390/s16030414

**Published:** 2016-03-22

**Authors:** Ning Li, José-Fernán Martínez, Juan Manuel Meneses Chaus, Martina Eckert

**Affiliations:** Centro de Investigación en Tecnologías Software y Sistemal Multimedia para la Sostenibilidad (CITSEM), Campus Sur Universidad Politécnica de Madrid (UPM), 28031 Madrid, Spain; jf.martinez@upm.es (J.-F.M.); juan.meneses@upm.es (J.M.M.C.); martina.eckert@upm.es (M.E.)

**Keywords:** underwater acoustic sensor network, routing protocol, cross-layer design, intelligent algorithm based

## Abstract

Underwater acoustic sensor networks (UASNs) have become more and more important in ocean exploration applications, such as ocean monitoring, pollution detection, ocean resource management, underwater device maintenance, *etc.* In underwater acoustic sensor networks, since the routing protocol guarantees reliable and effective data transmission from the source node to the destination node, routing protocol design is an attractive topic for researchers. There are many routing algorithms have been proposed in recent years. To present the current state of development of UASN routing protocols, we review herein the UASN routing protocol designs reported in recent years. In this paper, all the routing protocols have been classified into different groups according to their characteristics and routing algorithms, such as the non-cross-layer design routing protocol, the traditional cross-layer design routing protocol, and the intelligent algorithm based routing protocol. This is also the first paper that introduces intelligent algorithm-based UASN routing protocols. In addition, in this paper, we investigate the development trends of UASN routing protocols, which can provide researchers with clear and direct insights for further research.

## 1. Introduction

In the near future, the ocean will supply a substantial part of human and industrial needs: the oil and gas industry will move into deeper waters, renewable energy will be harvested from the sea, *etc.* Furthermore, minerals such as cobalt, nickel, copper, rare earths, silver, and gold will be mined from the seafloor. To this end, new offshore and port infrastructure will need to be built, maintained, and repaired, but ocean monitoring and research are not an easy task, since the ocean is big and most of the underwater environment is still unknown to us. In addition, due to the high pressures in deep water, it is not suitable for people to work a long time under water. Therefore, considering the wide applications of wireless sensor networks, researchers are trying to use wireless sensor networks to replace the traditional ocean exploration and monitoring methods. Since radiofrequency (RF) waves are seriously attenuated in the underwater environment [1], underwater sensor nodes use acoustic waves rather than RF wave to communicate with each other [2]. This kind of wireless sensor network is the underwater acoustic sensor network (UASN).

In UASNs, one of the hot research areas is routing protocol design. A routing protocol guarantees reliable and effective data transmission from the source node to the destination node. Considering the differences between the terrestrial and the underwater environment, UASN routing protocol design is more difficult and restricted than that of WSN [3]. First, the continuously movement of nodes with water currents makes underwater routing highly unreliable; second, the high propagation delay in the underwater environment is inefficient; thirdly, the special characteristics of underwater acoustic waves and channels limit the wide application of WSN technologies. In addition, underwater acoustic sensor networks are always deployed in the area where no arrangement in advance is possible, so the routing protocol should have the ability to build highly reliable and effective communication links without the help of pre-arranged devices. Moreover, in case the routing is broken during the data transmission, the routing protocol should able to repair or rebuild the routing in a timely way. The routing protocol must be robust and self-adaptive, which is very important for networks in harsh underwater environments.

A large amount of research has been carried out on the design and application of underwater acoustic sensor networks. These works have investigated various aspects of UASNs, such as the routing protocol, the MAC protocol, the link layer protocol, the communication method, the localization algorithm, *etc.* In [3], the authors introduced the principles and characteristics of different routing protocols, and compared different aspects of the different routing protocols, such as the data copies, the transmission method, clustering *vs*. non-clustering, single/multiple sinks, the control packets, *etc.* In addition, the authors also classified the different routing protocols based on the network architecture, the data forwarding method, and the protocol operation. The work in [4] reviews the state-of-the-art of the underwater acoustic sensor network protocol design, including the research methods of underwater acoustic sensor network and the research challenges of the different layers (MAC layer, network layer, and transport layer). A similar review can be found in [5]. In this paper, the authors provided a comprehensive overview of the current research on underwater acoustic sensor networks and analyzed the state-of-the-art of research on the physical, MAC and network layers.

Different from previous surveys, this paper focuses on the underwater acoustic sensor network routing protocol design. There are many kinds of communication protocols for underwater acoustic sensor networks, such as the MAC protocol, the topology control protocol, the link layer protocol, *etc.*, but that research is out of the scope of this paper and can be found in other relevant research articles. In this paper, we only consider the routing protocols of UASNs. Different from the already existing UASN routing protocol surveys, we divide the routing protocols into two different kinds: the cross-layer design routing and the non-cross-layer design routing. We investigate the research articles published in recent years and briefly analyze the performance of these routing protocols.

In addition, with the development of intelligent algorithms in recent years, more and more intelligent algorithms are being introduced into routing protocol design, especially in the applications which have high performance requirements. Intelligent algorithm-based routing protocols can achieve better performance than traditional routing algorithms, and have been widely used in the terrestrial environment, so even though an overview of intelligent algorithm-based routing protocols in the terrestrial environment is not the main scope of this paper, in order to provide an complete background for readers, we will introduce these routing protocols briefly.

The contributions of this paper are as follows:

- As the cross-layer design method has become more and more important in recent years, we classify the routing protocols based on the cross-layer design method and non-cross-layer design method. To the best of our knowledge, our survey is the first paper that provides a detailed classification based on cross-layer design methods.
- Considering that the intelligent algorithms can effectively improve the routing performance, we also review the intelligent algorithm-based routing protocols which can provide a wide range of concepts for routing protocol design.
- To give researchers clear and direct insights for the development of underwater acoustic sensor network routing protocols, in this paper, we investigate the development trends in UASN routing protocol design in recent years.

**Figure 1:**
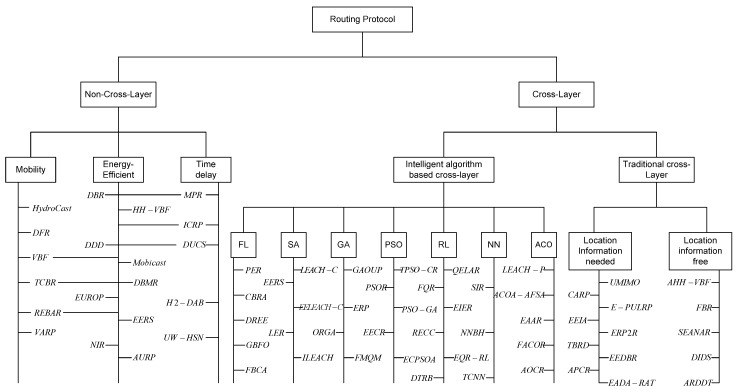
The classification of UASN routing protocols.

The classification of the UASN routing protocols in this paper is shown in Figure 1. The rest of this paper is arranged as follows: in Section 2, we will introduce the background knowledge of routing protocol design, including the principle of cross-layer design and different kinds of intelligent algorithms; in Section 3, we will introduce the knowledge of underwater acoustic sensor network briefly; in Section 4, we will review the traditional non-cross-layer design routing protocols; in Section 5, the cross-layer design routing protocols which include the traditional cross-layer design routing protocols and the intelligent algorithm based routing protocols will be investigated; in Section 6, we conclude the challenges and open issues of UASN routing protocol design, and discuss the developing trends of UASN routing protocols; finally, in Appendix, we compare different aspects of the routing protocols.

## 2. The Background of Routing Protocol Design

In this section, we will introduce the related principles of routing protocol design, such as the principle of cross-layer design, the difference between the cross-layer design and the non-cross-layer design, and the intelligent algorithms which can be used in routing protocols.

### 2.1. The Principle of Cross-Layer Design

In non-cross-layer routing protocol design, the network is divided into five layers and every layer can be designed and operated independently to achieve specific functions. The interfaces between different layers are static. The protocols for each layer can manage the issues in that layer based on the services provided by the lower layer, and can provide services to the upper layer. In this kind of modularized protocol stack, communication only occurs between adjacent layers. Different protocols are independent, and only need to describe the interfaces between different layers. Moreover, this kind of architecture has clear logic, good expansibility, high robustness, and is easy to implement [6,7].

Different from non-cross-layer design, cross-layer design is a method that shares the information with other layers. However, the cross-layer design does not abandon the layered structure completely; it just weakens the bounds and increases the interfaces between different layers. At present, there are many papers introducing the principles of cross-layer design, but there is still no uniform definition for cross-layer design. In this paper, we will reference the definition in [8] as the cross-layer design definition:

*Definition 1.*  The goal of cross-layer design is to achieve better network performance. The cross-layer design method should have the ability to preserve the layered architecture, break the communication limitations between different layers, and provide necessary QoS services for the network.

**Figure 2:**
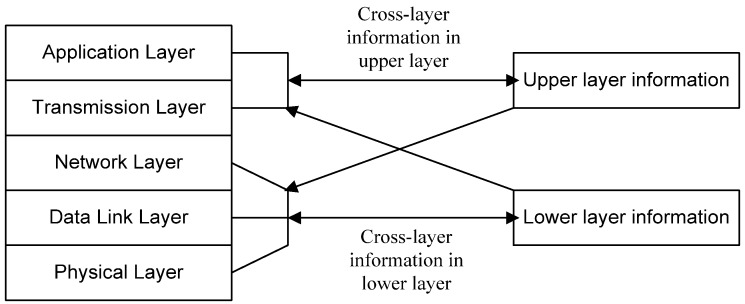
The principle of cross-layer design.

The cross-layer design can adjust the parameter configuration in different layers [9]. The principle of cross-layer design is shown in Figure 2. The main object of cross-layer design is to increase the information sharing in different layers and decrease the cost of communication and signal processing [9], *i.e.*, to meet the QoS requirements of wireless sensor networks.

**Figure 3:**
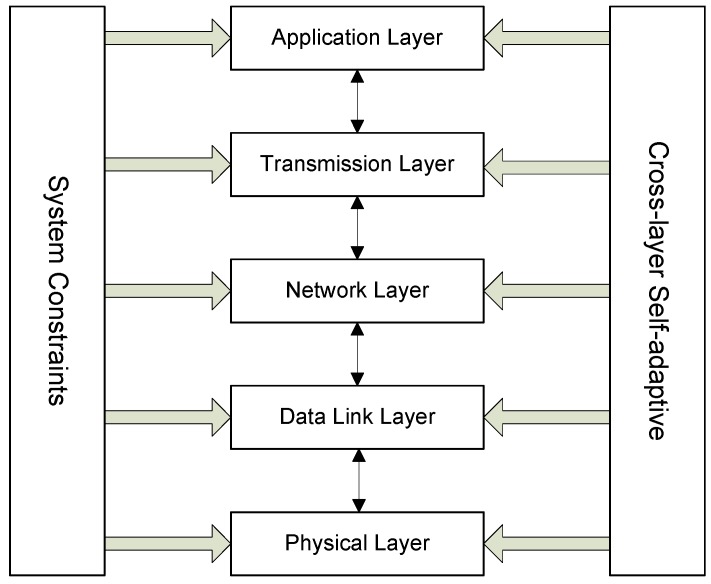
The architecture of cross-layer design.

Since the wireless channel is time-varying, during the routing protocol design, the abilities that allow the changing of wireless channels and meeting the QoS requirements must be taken into consideration, which are also the main objectives of cross-layer design. The term self-adaptive means that the protocol can adapt to changing environments and parameters to meet the requirements of energy consumption, network load, delivery ratio, *etc.* In traditional networks, these adjustments are processed in different layers independently; however, in cross-layer design, the different layers can exchange information with each other. The architecture of cross-layer design is shown in Figure 3.

The common theories and methods of cross-layer design include the optimal control, the intelligent algorithm based method, the dynamic programming, *etc.* At present, the technologies used in cross-layer design are as follows:

1. The scheduling technology. The scheduling technologies include node access scheduling, link utilization scheduling, network application scheduling, resource reserve, and data transmission priority allocation. The scheduling technology can relax the burstiness of network flow and make the system more appropriate for changing networks [10,11].
2. The diversified technology. The diversified technologies include link characteristic diversity, routing chosen diversity, application requirement diversity, and access technology diversity, *etc.* The diversity can enhance the system capability to adapt the network dynamics and improve the network reliability [12,13,14].
3. The self-adaptive mechanism. The term self-adaptive means the protocols and the applications have the ability to adapt to changing channel conditions and network topologies. The self-adaptive mechanisms include link layer adaptive, network layer adaptive, and application layer adaptive. By cooperating with the diversified technology and the scheduling technology, the self-adaptive mechanism can greatly improve the system robustness [15,16].

### 2.2. The Intelligent Algorithm

Since intelligent algorithms are much more effective than traditional algorithms, especially in complex and non-linear problems, intelligent algorithms become more and more attractive in wireless sensor network protocol design. Many intelligent algorithms were proposed in recent years. However, considering their computing features, not all intelligent algorithms can be used in wireless sensor networks. In this paper, we review the research articles in recent years and find some available intelligent algorithms for routing protocol design. These intelligent algorithms include Simulated Annealing (SA) [17], Genetic Algorithm (GA) [18], Ant Colony Algorithm (ACO) [19], Particle Swarm Optimization (PSO) [20], Reinforcement Learning (RL) [21,22], Fuzzy Logic (FL) [23], and Neural Network (NN) [24]. In addition, in [25], the author also introduces the principle of SA. The detailed introduction of these intelligent algorithms is beyond the scope of this paper and can be found in the corresponding references.

## 3. The Knowledge of Underwater Acoustic Sensor Networks

Since an underwater acoustic sensor network is used to collect data when events occur in the underwater environment, a reliable and effective route from source node to the destination node is necessary. Even though many routing protocols have been proposed for terrestrial wireless sensor networks, considering the differences between the underwater environment and the terrestrial environment, terrestrial routing protocols are different from UASN routing protocols. For better understanding the differences between underwater communication and terrestrial communication, and the difficulties in routing protocol design for underwater acoustic sensor networks, some investigation of the characteristics of underwater acoustic communication is necessary.

### 3.1. The Characteristics of Underwater Acoustic Communication

Due to the different transmission media, the underwater environment poses a more severe situation for routing protocol design than terrestrial wireless sensor networks. In the following paragraphs of this section, we will introduce these differences briefly.

#### 3.1.1. High propagation delay

Underwater sensor nodes use acoustic waves rather than RF waves to communicate with each other. As shown in [26], the speed of RF waves in air is about 200,000 times faster than acoustic waves under water, so the propagation time in underwater acoustic sensor networks is appropriately 200,000 times longer than in terrestrial wireless sensor networks, which will cause serious propagation delays. In addition, changing salinity, temperature, and depth all can affect the propagation speed of underwater acoustic waves. The propagation delay in underwater environments is dynamic, which also increases the difficulty of routing protocol design.

#### 3.1.2. High energy consumption

The acoustic wave attenuation in underwater environments is serious. The transmission power needed by the underwater acoustic transceivers is in order-of-magnitude higher than that of terrestrial devices [27]. In addition, due to the continuous movement of underwater nodes, the communication links are easily broken. Moreover, the bit error rate in underwater acoustic sensor networks is much higher than in terrestrial wireless sensor networks. All these issues can lead to frequent data packet retransmission, which will waste vast amounts of energy.

#### 3.1.3. Low bandwidth and data rate

The bandwidth of acoustic waves depends on the transmission distance. The bandwidth ranges from 1 kHz to 50 kHz [28]. In addition, considering the fact that acoustic waves have high power absorption in the underwater environment, only a few frequencies can be used to communicate over a long distance. The frequencies range that the acoustic wave can work under the water is from a few Hz to tens of kHz, thus the transmission rate can hardly exceed 100 kbps which cannot compare to RF waves in air. Therefore, this represents a great limitation in UASN routing protocol design, especially considering that the routing protocols need large amounts of information exchange during routing discovery and routing maintenance.

#### 3.1.4. High noise and interference

Due to the water currents, the machines, and the ships in the water, the noise under water is much more serious than in the terrestrial environment. The interference under the water is also higher than in the terrestrial environment, which is mainly due to the reflections caused by the surface, the bottom, or animals and impurities in the water. In addition, the underwater refraction is serious, too, so the multipath interference in underwater acoustic sensor networks is more serious than in terrestrial wireless sensor networks.

#### 3.1.5. Highly dynamic topology

In the terrestrial environment, once the sensor nodes are deployed, they cannot move around easily and frequently, so the topology changes only when old nodes die or new nodes join the network. However, in the underwater environment, due to the water currents, the sensor nodes are continuously moving with the water currents, therefore, the topology changes frequently, which has a great effect on the routing protocol performance.

The differences between the underwater environment and the terrestrial environment are shown in Table 1. Due to the great differences, the standard wireless sensor network routing protocols cannot be applied to UASNs directly. We need to redesign or modify the WSN routing protocols according to the underwater environment conditions to meet the QoS requirements of UASNs.

### 3.2. The Energy Consumption

No matter whether in a terrestrial wireless sensor network or an underwater acoustic sensor network, the energy consumption is always the first important factor, since low energy consumption means long network lifetime. Many energy consumption models have been proposed in recent years. In [29], the authors proposed a realistic power consumption model for WSN devices by incorporating the characteristics of typical low power transceivers. In [30], an energy model that is specifically built for online accounting is presented. This model only considers two states of the microcontroller and radio chip. The energy model in [31] uses a set of finite state machines to represent the states and transitions of a sensor node’s hardware. There are also many energy consumption models that can be found in [32,33,34,35]. For instance, in [35], the authors propose an energy consumption model for underwater wireless sensor network in which both shallow water conditions and deep water conditions are taken into account. Even through these energy consumption models are all proposed for terrestrial wireless sensor networks and cannot be applied directly in underwater environments, the review of these models can provide some guidance for underwater wireless sensor network energy consumption analysis.

### 3.3. The Propagation Delay

Due to the fact the propagation delay varies with different scenarios and applications, up to now, there is no uniform model or standard for the propagation delay of underwater sensor networks. Therefore proposing an available model or standard for underwater acoustic sensor network propagation delay is an open issue and a challenge for further research. The propagation delay of underwater sensor networks is mainly related to the communication media properties and the network scenario environment. The propagation speed changes frequently with temperature, depth, and salt density. In addition, considering the moving speed of AUVs and sensor nodes, this propagation delay will have great effect on the routing protocol performance; sometimes, it can disable the routing, so how to calculate the propagation delay in underwater acoustic sensor networks is important and necessary for routing protocol design.

### 3.4. The Movement of Underwater Sensor Nodes

Another challenge of the routing protocol design is the continuous movement of underwater sensor nodes. As we know, different motion models have different effect on the same routing protocol, so when we design the routing protocol, we must take the mobility of the underwater sensor node into consideration. There are already many motion models for terrestrial sensor nodes [36], such as the random walk mobility model, the random waypoint mobility model, the random direction mobility model, and the Guess-Markov mobility model. However, these motion models mainly applied in 2-D network architecture, but the network is 3-D in the underwater environment, so these motion models cannot be directly applied in underwater wireless sensor networks. The research on hydrodynamics shows that the movement of underwater objects closely relates to many factors, such as the water current and the water temperature. In different environments the motion models are different. For instance, the movement behaviors are different in a river and near the seashore. However, as discussed in [37,38], the movement of underwater objects is not a totally random process. Temporal and spatial correlation is inherent in such movement, which makes their mobility patterns predictable. In [39], the authors propose a motion model for underwater sensor networks.

## 4. The Non-Cross Layer Design Method

In this section, we briefly introduce the traditional non-cross-layer design UASN routing protocols. In recent years, cross-layer design UASN routing protocols have been widely used and investigated. The traditional non-cross-layer design UASN routing protocols are the basis of cross-layer design UASN routing protocols. The cross-layer design routing protocols are mainly modifications or improvements based on non-cross-layer protocols. Therefore, a review of traditional non-cross-layer routing protocols is necessary for the development of UASN routing protocols.

The underwater acoustic sensor network routing protocols are devoted to providing effective and reliable routing from the source node to the destination node. As discussed in Section 3, in the underwater environment, the propagation speed of sound waves is much slower than RF waves, *i.e.*, the propagation delay in UASNs is serious. In addition, in the underwater environment, sound wave attenuation is much more serious than that of RF waves. These problems will become more serious in underwater acoustic sensor networks which are sparse networks (the distances between two nodes are always long). Moreover, underwater sensor nodes are usually powered by batteries and their energy is limited, so how to reduce the energy consumption in underwater acoustic sensor networks is the first important issue for routing protocol design. Almost all routing protocols take the energy consumption into account. In addition, due to the mobility of underwater sensor nodes, the frequently changing network topology will decrease the routing reliability. This mobility therefore should also be considered in routing protocol design to increase the routing reliability. In the following of this section, we will review these routing protocols based on three factors: energy consumption, propagation delay, and node mobility.

### 4.1. The Energy Efficient Routing Protocol

Since underwater sensor nodes are always deployed in harsh environments and powered by batteries, the total energy is strictly limited. In addition, it is not appropriate to change batteries for underwater sensor nodes (sometimes this is impossible). Consequently, the first important issue of an underwater sensor network is improving the energy efficiency to prolong the network lifetime. Many UASN routing protocols have been proposed to improve the energy efficiency performance, such as those described in [40,41,42,43,44,45,46,47,48,49].

Due to the fact that depth information is much easier to get than location information in underwater environments, many routing algorithms utilize depth information instead of location information to reduce the energy consumption and propagation delay. In [40], the authors propose depth-based routing (DBR), which use depth information to reduce the invalid broadcasts. The nodes are equipped with depth sensors to calculate the node’s depth. Based on the depth information, the data packets are transmitted to the sink node. Considering that the transmission model in DBR is flooding, in the depth-based multi-hop routing protocol (DBMR) [45], a multi-hop transmission model other than the flooding model is applied to reduce the energy consumption. Like DBR and DBMR, in the energy efficient routing protocol (EUROP) [42], the nodes in the network are divided into different layers based on the depth information. The nodes in the same layer can communicate with each other. The nodes can move to the surface and back to their pre-defined place. The data packets are transmitted from the deep water to the shallow water. Even these depth information-based routing algorithms can reduce energy consumption and propagation delay successfully, however, the nodes must be equipped with special sensors to calculate the depth information, which is expensive and can introduce extra energy consumption into the nodes.

Aside from depth information, in some routing algorithms, AUVs or mobile nodes are used to improve the routing performance and data collection efficiency. In the mobile delay tolerant approach (DDD) [41], the authors use dolphin nodes which can move randomly or in a preplanned way to collect the data that is stored in fixed nodes. The fixed nodes use a low power communication model to detect the dolphin nodes and transmit data to the dolphin nodes through a normal transmission model. Different from DDD, in Mobicast [44], the nodes near the AUV or mobile sink can form a 3-D geographic zone. The AUVs or the mobile sinks travel along a user-defined route and continuously collect data from the nodes that are within the 3-D zone. Similar routing protocols can also be found in [47,48]. In [47], the sensors send data to GWs, and the GWs send data to AUVs when the AUVs pass through the GWs. The AUVs can send normal data to sink nodes through acoustic channels and urgent data to satellites through RF channels. In temporary cluster-based routing (TCBR) [48], the nodes near the sink node can achieve balanced energy consumption with the help of mobile nodes. The energy consumption in AUV aided routing algorithms can be reduced seriously: (1) most nodes are fixed and can go to sleep when the AUVs are out of their transmission range; (2) the communication between the AUVs and the fixed nodes uses a low cost communication model. The disadvantage is that the travel route, the number, and the transmission ranges of AUVs all can affect the routing performance. In addition, the propagation delay of the AUV aided routing algorithm cannot be ignored.

Clustering is an efficient method to reduce energy consumption and propagation delay in underwater acoustic sensor networks. In [49], the authors propose a distributed underwater clustering scheme (DUCS). The DUCS divides the nodes into different clusters for reducing energy consumption; the communications between nodes are divided into inter-cluster and intra-cluster, which is efficient. However, in DUCS, the authors do not take the node mobility into account, so when the nodes move continuously, the performance is not as good as in the simulation.

In [46], the authors study the effects of the channel characteristics on energy consumption and time delay under different path distances and hop lengths. Based on the results, the authors propose a set of geographic information-based routing protocols which are energy efficient.

In this section, the energy efficiency routing protocols are introduced. The works in [40,42,43,45] use depth information to decrease the unnecessary energy consumption. The energy consumption in [45] is smaller than that in [40], since the transmission method is multi-hop in [45] rather than flooding as in [40]. The routing performance in [42] is better than that in [40,45]; however, in [42], the nodes are equipped with extra devices, which is expensive. Even mobile nodes are used in [41,44,47,48], and these routing protocols have small energy consumption, but these protocols have serious propagation delays which are not appropriate in the real time applications. The cluster-based routing protocol introduced in [49] has better performance than the depth information and mobile node-based routing protocols, but the work in [49] cannot deal with the issue of node movement, which deteriorates the routing performance.

### 4.2. The Mobility

In this section, the mobility of underwater sensor nodes caused by water currents is taken into consideration by the routing protocols. Due to the continuous movement of the sensor nodes, the routing is easily broken, which will need extra energy and processing time to recover or rebuild the routing, so the routing protocols which take the node mobility into consideration also have good energy consumption and propagation delay performance.

**Figure 4:**
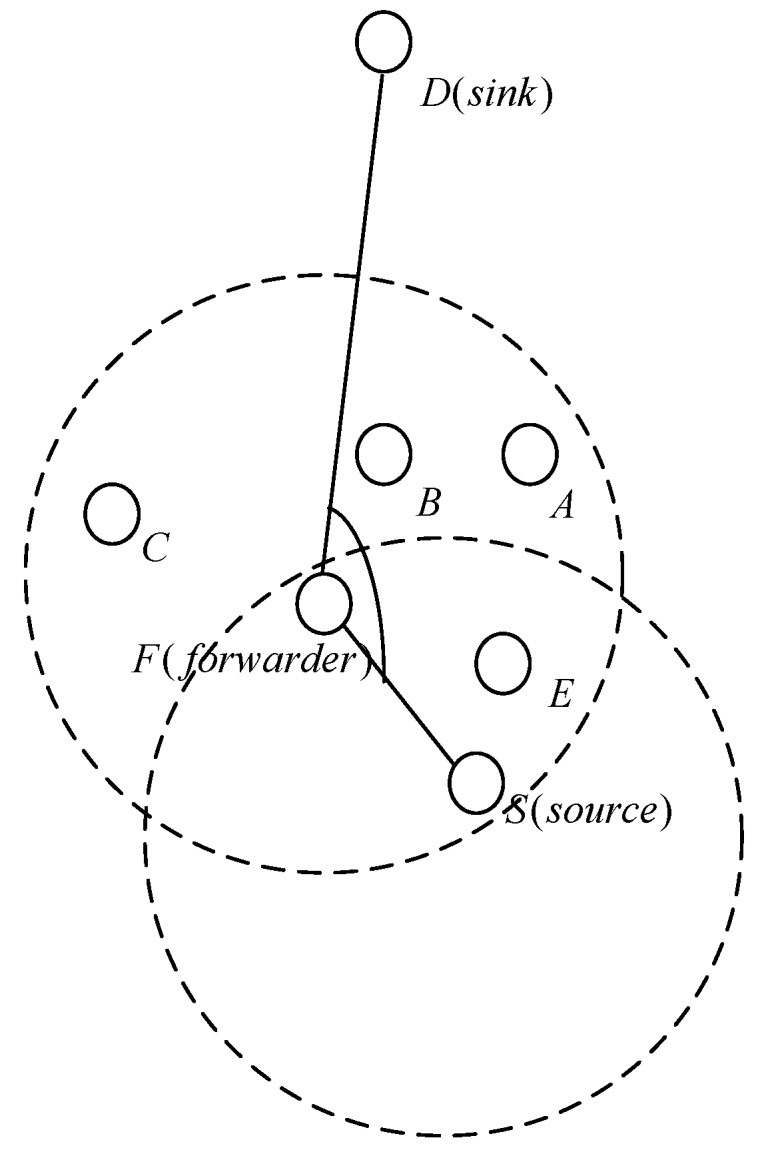
The principle of DFR.

Since the node mobility affects the link quality, some mobility aware routing algorithms take the link quality into account to improve the network performance. In [50], considering the unreliability of acoustic channels, the authors propose HydroCast. HydroCast is similar with DBR, the difference beingthat HydroCast take the wireless channel quality into consideration to improve the routing performance under continuous node movement conditions. The routing algorithm introduced in [51] also takes the link quality into account to increase the reliability of the communication links. By defining the flooding zone, the routing protocol can adapt to the dynamic changing of the network conditions and topology, see Figure 4. However, the disadvantages in these two algorithms are obvious: (1) in HydroCast, the authors do not consider the energy consumption of each node; (2) in [51], the nodes need to know their own location information and that of the destination node, which is not always available in underwater sensor networks.

Considering the frequent routing maintenance and discovery in mobile UASNs, the authors in [52] propose a vector-based forwarding (VBF) routing protocol. In VBF, a virtual routing pipe is used to forward data packets to destination nodes. The nodes located in the pipe can take part in the data transmission. The routes from the source node to the destination node are redundant and separate, which can make the routing protocol robust to node movement. However, in VBF, the nodes near the pipe will be used again and again, which is not energy balanced. To overcome this problem, in [53], the authors propose a hop-by-hop vector-based forwarding protocol (HH-VBF which has better performance than VBF. In HH-VBF, each node can decide the pipe direction based on the current node location. However, due to the hop-by-hop transmission method, the overheard is heavier than in VBF; moreover, the performance of HH-VBF is sensitive to the pipe radius threshold.

The interesting conclusion in [54] is that the authors regard the continuous node movement as having a good effect on energy balance. Based on this conclusion, the reliable and energy balanced routing (REBAR) algorithm is proposed. In REBAR, the nodes have different transmission ranges according to their distances to the sink node. The nodes near the sink node have small transmission ranges, and *vice versa*. The algorithm can reduce the energy consumption of nodes near the sink node. However, in fact, node movement can cause more problems than advantages. Void aware pressure routing (VAPR) [55] is also a robust routing protocol for dynamic topology networks, in which the node uses sequence number, hop count, and depth information to set up the next hop relay node.

In this section, three different kinds of mobility-aware routing protocols are reviewed. The work in [50,51] considers the link quality in the routing protocol design. As for the node movement, the work in [51] can achieve better performance than HydroCast. However, in HydroCast, the authors do not consider the energy consumption of each node; in [51], the nodes need know their own location information and that of the destination nodes, which is not always available in underwater sensor networks. VBF and HH-VBF are routing protocols which can successfully reduce the routing rediscovery and maintenance. The HH-VBF has better performance on the energy consumption balance than VBF, but the overheard of HH-VBF is heavy. The idea in [54], which states that node movement has a good effect on energy balance is novel; however, the real performance of REBAR is not as good as the theoretical analysis suggests.

### 4.3. The Propagation Delay

Due to the high attenuation ratio and low propagation speed of acoustic waves in the underwater environment, the propagation delay is a serious problem in UASNs. Reducing the propagation delay also can reduce the energy consumption. Routing protocols which have small propagation delays also have good energy consumption performance.

In underwater acoustic sensor networks, one cause of propagation delays is that the propagation speed of acoustic waves is slow, so in [56], the authors propose underwater wireless hybrid sensor networks (UW-HSNs). In UW-HSNs, the communication is divided into two parts: radio communication (which is used to communicate with the base station on the surface) and acoustic communication (which is used for underwater communication with other nodes). The UW-HSN is effective and has small transmission delays. However, the nodes in UW-HSN must be equipped with both radio communication modules and acoustic communication modules, which is not energetically optimal.

Considering the propagation delay in routing discovery and data transmission, the hop-by-hop dynamic addressing-based routing protocol (H2-DAB) [57] and information carrying routing protocol (ICPR) [57] are proposed. In H2-DAB, the data packets are transmitted hop-by-hop; the algorithm does not need any special devices or information to build the routing path. A novel routing algorithm named ICPR has been proposed in [58], where the routing discovery and data transmission are processed simultaneously, *i.e.*, transmitting data packets while building the routing. ICPR is time saving and can greatly reduce the propagation delay. In [59], a routing protocol named low propagation delay multi-path routing protocol (MPR) is proposed. In MPR, all the nodes can send data packets to surface sink nodes; the source nodes divide the data packets into several time slots based on the bandwidth and use a two-hop transmission scheme to transmit data packets to the relay nodes. In MPR, multi-path is utilized during the path construction from the source node to the destination node, which can avoid data collisions in receiving nodes.

In this section, two methods are used to reduce the propagation delay: one is to separate the overwater communication and the underwater communication [56]; another one is to reduce the processing time in routing discovery and data transmission [57,58,59]. All these routing protocols have good propagation delay performance, but the problem is that the energy consumption in these routing protocols is much higher than in other routing protocols due to the heavy overhead [57,58,59] or the high energy consumption in the radio communication module.

### 4.4. Summary

In Section 4.1, Section 4.2 and Section 4.3, we have reviewed the traditional non-cross-layer routing protocols for underwater acoustic sensor networks. These routing protocols relate to the energy consumption, the propagation delay, and the mobility, which are also the main factors of routing protocol design.

For overcoming the weakness that the energy consumption of an underwater acoustic sensor network is much higher than that of a terrestrial wireless sensor network, energy-effective routing protocols are proposed. These routing protocols use the location information or the depth information to reduce unnecessary broadcasts and hop counts to save energy, such as in [40,42,45]; or work with the assistance of AUVs, such as in [41,44,47].

The propagation delay is another factor which can affect the routing performance. As analyzed in Section 3, the reasons for propagation delay are the low transmission speed of acoustic waves and the unreliable underwater acoustic channel, so approaches used to reduce propagation delay mainly concentrate on three aspects: first, using location information to reduce unnecessary routing discovery; second, reducing the distance between two relay nodes; third, reducing retransmissions as much as possible. The mobility also has a great effect on routing performance. To this end, the location information and the hop-by-hop transmission model are used to reduce the routing maintenance and routing rebuilding, which can obviously reduce the energy consumption and propagation delay.

## 5. The Cross-Layer Design Method

The cross-layer design method has become more and more important and common in recent years. Research on cross-layer design mainly relates to two methods: the optimization-based cross-layer method and intelligent algorithm-based cross-layer method. In the rest of this section, we will discuss these two methods in detail.

### 5.1. The Optimization-Based Method

As discussed in Section 2, the cross-layer design methods can be abstracted as a series of optimization issues. The parameters that are considered in cross-layer design methods are energy consumption, propagation delay, throughput, transmission rate, and so on. Due to the fact that we cannot get optimal solutions for all factors at the same time, the traditional cross-layer methods cannot deal with the NP-hard problems. Unfortunately, many optimizations of the cross-layer parameters are NP-hard issues, such as the minimum energy consumption and the maximum throughput, the minimum energy consumption and the highest transmission rate, *etc.* However, there are still many cross-layer optimization issues that can be solved by cross-layer methods.

Since the cross-layer routing protocols have the capability to handle more than one cross-layer parameter, we cannot distinguish the cross-layer routing protocols based on the energy consumption, the propagation delay, or the transmission rate, *etc.* In this part, we classify the traditional cross-layer design routing protocols based on whether the location information is needed.

#### 5.1.1. Location Information-Free Routing Protocols

The location information free routing protocols do not need node location information. Due to the absence of location information, these routing protocols use the flooding model, the deep information, or the hierarchy model to send data packets from the source node to the sink node. Due to the fact that getting location information in the underwater environment is much more difficult than that in the terrestrial environment, in underwater environments, the routing protocols always assume that the location information is unknown. However, without the help of the location information, the routing discovery process is always blind, which increases the energy consumption and propagation delay. The challenge of location information free routing protocols is how to reduce this blindness and improve the algorithm efficiency.

There are many papers devoted to the design of cross-layer design routing protocols without utilizing node location information, such as [60,61,62,63,64,65,66,67,68]. The routing protocol in [60] uses the history of successful transmission to the neighbor nodes, the link quality, the residual energy, and the buffer space to select next hop relay nodes. For this reason, the protocol has good next hop node selection and energy consumption performance. In [62], the nodes are divided into different levels based on their residual energy, only the nodes in the same energy level can be chosen as a next hop relay node. The work in [63] is also an energy efficient routing protocol. In [63], each node can decide a specific forwarding group rather than all the neighbor nodes to reduce unnecessary broadcasts and transmissions. The relay nodes are chosen based on the distance to the sink node and their residual energy, which can successfully prolong the network lifetime. Like [62], the work in [65] divides the nodes into different levels based on their power level. The nodes can adapt their transmission power based on the network conditions. The power level and the residual energy of nodes are used to choose the next hop relay nodes. The proposal in [66] is similar to the protocol introduced in [62,65]. In [66], the depth information and the residual energy of nodes are chosen to decide the next hop relay nodes. The nodes whose depth is smaller than the source node and whose residual energy is higher will be chosen. The result is that the network lifetime can be prolonged when using [66]. A similar routing protocol can be found in [64], which proposes a set of routing protocols and scheduling to reduce the effect of interference and energy consumption.

Some papers not only consider the distance and residual energy, but also combine the network constraints with other methods to improve the network performance. In [61], the authors introduce MIMO-OFDM (Multiple-Input Multiple-Output Orthogonal Frequency Division Multiplexing) into underwater acoustic sensor network routing protocol design, which successfully achieves excellent energy consumption and propagation delay performance. The work in [67] is a tier-based UASN routing protocol. In this routing protocol, the network topology is partitioned into tiers, and the next hop relay nodes towards the surface sink are limited to the nodes that belong to the upper tier. The protocol in [68] is an adaptive tree reconfiguring protocol where the data aggregation tree is reconfigured by using a dynamic pruning and grafting function. The function can change the routing path based on the information of the aggregation count and minimum residual energy of nodes.

In this section, cross-layer design location information-free routing protocols are reviewed. In cross-layer design methods, more than one network constraint can be considered in routing protocol design. The most common factors are depth information [63,66,67] and the residual energy [60,62,65,68]. The residual energy-based routing protocols have good energy consumption performance, and the depth information-based routing protocols can achieve good propagation delay performance. The performance of [63,67] are similar and they are both better than [66], since the works in [63,67] take both the depth information and the residual energy into account. The work in [60] has better performance than the other routing protocols, because in [60] the link quality, the buffer space, and the history of successfully transmission are all taken into account. The performance of [62,65] which divide the network into different layers based on the residual energy is similar, but the propagation delay in these two protocols is worse than in [63,66,67].

#### 5.1.2. Location Information-Based Routing Protocols

With the development of underwater GPS and underwater location algorithms, it is attractive that the routing protocols utilize the location information to reduce the blindness in routing discovery, which can save energy and reduce propagation delay remarkably. As we know, if the source nodes know the location of other nodes (such as the sink node and the relay nodes), the process of routing discovery will be more effective than that of the location information-free routing protocols. Even though location information-based routing protocols are effective, considering the expensive underwater GPS devices needed and the complexity of the underwater location algorithm, the performance of the location-based routing protocols will decrease. The challenge is to find a more simple approach to get the location information of nodes.

**Figure 5:**
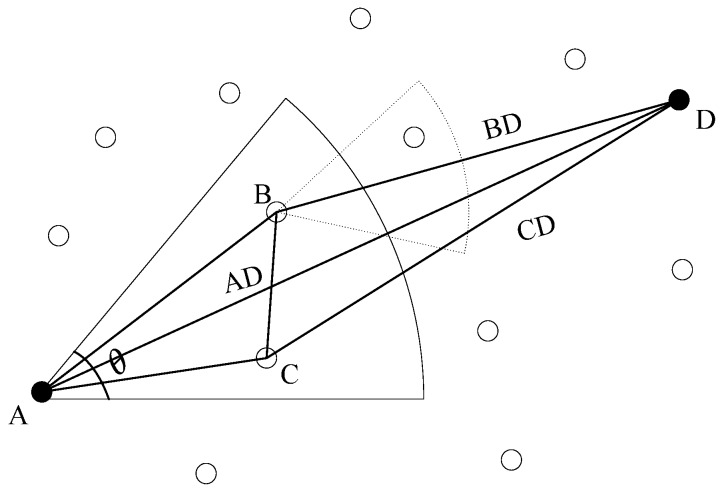
The principle of FBR.

The routing protocol proposed in [69] is based on HH-VBF [43], but by taking the power level, the distance to the destination node, and the transmission range into account, the performance of adaptive hop-by-hop vector-based forwarding (AHH-VBF) [69] is much better than that of HH-VBF in terms of data delivery ratio, energy consumption, and end-to-end latency. Considering that greedy flooding is energy intensive, in the focused beam routing (FBR) protocol [70], the authors try to use the location information of source nodes and destination nodes to build the routing path. To improve the routing robustness, the nodes can dynamically change their transmission power according to the network conditions. FBR can achieve better energy consumption performance, but it is not flexible and appropriate for sparse networks. The principle of FBR can be found in Figure 5. In [71], the protocol relies on the degree of information of neighbor nodes to make routing decisions. The nodes that have different node degree belong to different layers. The node ID, the coordinate information, and the residual energy level are used to decide the node degree. The information can only be transmitted from low level degree to high level degree nodes. The authors in [72] investigate the function between the corresponding node distance and packet size. Based on this function, two geographical routing algorithms are proposed. The algorithms take the transmission power, the channel quality, and the application requirements into account to choose the next hop relay nodes. Realizing the fact that an underwater sensor network is an intermittently connected network and different applications have different requirements, in [73], the authors propose a routing protocol in which the routing is performed adaptively based on the message types and application requirements. The algorithm allows packets with higher priority to forward more copies. The routing decisions are made based on the packet priority afterwards. As a result, the algorithm can not only satisfy different application requirements, but also achieve a good trade-off between delivery ratio, average end-to-end delay, and energy consumption.

In this section, routing protocols that introduce the need for location information to improve the routing performance were discussed. The location-based routing protocols have better performance than the location information-free routing protocols, so the performance in [70,71,72,73] is much better than that of the routing protocols that were introduced in Section 5.1.1. The reason is that once the nodes know their own location of that of the destination node, the routing discovery and data transmission will be less blind and more effective than in the location information-free routing protocols. However, the disadvantage is that the location information of underwater nodes is hard to get, and sometimes it is impossible in an underwater environment.

#### 5.1.3. Summary

In this subsection, we have introduced two different kinds of traditional cross-layer design routing protocols: location information-free routing protocols and location information-based routing protocols. As shown in Section 5.1, the location information-free routing protocols do not need node location information. These protocols use flooding, depth information, or hierarchy models to transmit data packets from the source nodes to the destination nodes. From Section 5.1, we can conclude that the disadvantage of location information-free routing protocol is that they are blind in routing discovery due to the absence of location information, which will waste vast amounts of energy and cause serious propagation delays. Unlike the location information-free routing protocols, the location information-based routing protocols can utilize the location information of nodes to assist the routing discovery. The location information can be obtained by underwater GPS or location algorithms. The challenge of location information-based routing protocols is that underwater GPS is expensive and energy intensive; moreover, the location algorithms are usually complex, which will waste large amounts of node resources.

### 5.2. Intelligent Algorithm-Based Methods

Even though the traditional cross-layer methods can greatly improve the routing performance, considering their limited capability for dealing with multiple constraints and high calculation complexity, the wide application of traditional cross-layer method is limited, so intelligent algorithm-based methods have been proposed to extend the application range of cross-layer methods.

The intelligent algorithm-based methods for cross-layer routing protocol design are still a new research area for underwater acoustic sensor networks. In recent years, many researchers have devoted their efforts to combine the traditional cross-layer design method and the intelligent algorithms together to find an effective approach for cross-layer routing protocol design. There are many excellent intelligent algorithm-based routing protocols have been proposed for terrestrial wireless sensor networks; however, only the fuzzy logic-based routing protocols have been applied in underwater acoustic sensor networks. Therefore, the review on these intelligent algorithm-based cross-layer design routing protocols should open our minds and provide more research methods for underwater acoustic sensor network routing protocol design.

#### 5.2.1. Fuzzy Logic-Based Routing Protocols

Fuzzy logic is a kind of science based on multiple-valued logic. Fuzzy logic utilizes a fuzzy set to represent fuzzy thinking, fuzzy language, and fuzzy control laws. In 1965, the mathematician Zadeh first proposed the principle of fuzzy sets [23]. The essential of fuzzy mathematics and fuzzy logic is to provide accuracy descriptors for fuzzy objects. Fuzzy logic uses mathematical methods to imitate the actions of the human brain, such as the reasoning mode, uncertainty analysis, *etc.* By using fuzzy sets and fuzzy rules to reason the transitivity limitations or qualitative experiences, fuzzy logic algorithms can imitate the human brain to achieve fuzzy logic judgment and resolve issues which cannot be addressed by traditional methods. Fuzzy logic is good at dealing with issues in which the limitations are not clear.

Many underwater sensor network routing protocols have introduced fuzzy logic algorithms into the protocol design [74,75,76,77,78]. In [74], the power efficient routing (PER) protocol includes two modules: the forwarding node selector and the forwarding tree trimming mechanism. The forwarding node selector uses a fuzzy logic inference system and decision trees to choose the next hop relay node. Three parameters are used as the inputs of the fuzzy logic inference system: the transmission range, the include angle, and the residual energy. Different from [74], the algorithms in [75,78] are cluster-based fuzzy logic routing algorithms. In [75], the authors propose a cluster-based fuzzy logic routing algorithm with even load distribution for large scale networks. This algorithm is similar to LEACH; the difference is that this protocol does not include TDMA scheduling formation in the initial setup phase. Based on these, the proposed protocol can prolong the network lifetime by an average 40% to 50% more than LEACH. In [78], taking the residual energy, the distance to sink node, the node density, the load, and the link quality into account, the authors propose a fuzzy logic-based clustering and aggregation technique for underwater acoustic sensor networks. The work in [77] is a grid-based fuzzy logic optimized routing protocol. In [77], the network is divided into different virtual grids. Each grid has only one active node. The fuzzy logic system is used to select the active node. In [76], the authors use the distance to the sink, the residual energy, and the link quality of the potential neighbors near the sink as the inputs of the fuzzy logic system to choose the best routing path, which has more balanced energy performance than the traditional routing protocols.

#### 5.2.2. Simulated Annealing Based

Simulated annealing was proposed by Metropolis in 1953 [17]. In 1983, simulated annealing was introduced into combinatorial optimization [25]. Simulated annealing is a kind of stochastic searching optimization algorithm based on a Monte-Carlo iterative solving strategy. The idea of simulated annealing is inspired by the similarity between the annealing process and combinatorial optimization. Simulated annealing starts with a high initial temperature; with the decrease of the temperature, the simulated annealing algorithm uses probabilistic jumping to find the optimal solution in solution space randomly, *i.e.*, the algorithm can jump between the locally optimal solutions probabilistically and finally get a globally optimal solution. The simulated annealing algorithm is a universal optimization algorithm which can theoretically achieve global optimization performance. The simulated annealing algorithm has been widely used in control engineering, machine learning, neural network, signal processing, *etc.*

Many researchers have used simulated annealing algorithms to improve the performance of LEACH, such as [79,80,81,82]. The algorithm shown in [79] uses a simulated annealing algorithm to form clusters. In low-energy adaptive clustering-centralized (LEACH-C) [79], the NP-hard problem of finding *k* optimal clusters has been solved. Based on LEACH-C, in [80], the authors propose an energy efficiency optimized LEACH-C algorithm for WSNs. To improve the energy performance, energy efficiency optimized LEACH-C takes packet retransmission and acknowledgement into consideration to create a cluster head energy consumption model. The algorithm can calculate the quadratic sum of the distances from each cluster head to its member nodes in the optimal solution. Based on LEACH, in [82], the authors use the member nodes’ information in steady phase to choose the vice cluster heads which will take over the role of cluster heads in the later steady phase period to avoid the early death of cluster heads. The work in [81] addresses the energy efficient sensor deployment problem. In this algorithm, a simulated annealing algorithm will be used to calculate the global optimization result for the whole network. In [83], the authors design a simulated annealing-based algorithm for large scale wireless sensor networks. In this algorithm, the Wiener Index is used as the cost of the solution. The algorithm randomly selects a neighborhood as the current solution and calculates the Wiener Index. If the Wiener Index is greater than or equal to the current solution, then the current solution will be replaced by the neighborhood. The algorithm has better energy efficiency and network latency performance.

#### 5.2.3. Genetic Algorithm-Based Approaches

The genetic algorithm, proposed by Holland in 1975 [18], is motivated by biological evolution theory. For the process of biological evolution in the natural world, living things can adapt to the changing environment according to natural selection mutation and genetic evolution. Based on this, researchers use biological evolution to simulate the searching and optimization process. In genetic algorithms, the points in the search space are utilized to represent the point, the object functions represent the ability of biological individuals to adapt to the environment, and the process of good solution replacing bad solutions in optimization and searching represents the process of survival of the fittest. The algorithm can select individuals based on a fitness value in search space and use the biological evolution theory to generate a new population which represents the new searching solutions. This process is similar to biological evolution, in which the offspring populations are much better than the previous generation in adapting to their environment. This property can be used in other research areas to find approximate optimum solutions.

As proposed in the genetic algorithm inspired routing protocol (GAOUP) [84], the genetic algorithm and simulated annealing are used to enhance the network performance. The GAOUP has great advantages in development time; moreover, the systems are robust and relatively insensitive to noise. In [85], to increase the stable period in cluster-based routing protocols, the evolutionary-based clustered routing protocol (ERP) uses a population of individuals that evolve toward maintaining an optimal number of clusters. Binary tournament selection is used in this algorithm; the selection process chooses the candidate individuals from the population in the current generation based on their fitness values. Even though the ERP can prolong the network lifetime and reduce the energy consumption, but stability awareness performance is poor. The protocol introduced in [86] is used to solve the shortest path routing problem. The algorithm uses the genetic algorithm to select the best route from a set of random routes based on the fitness values of each route. In [87], the authors proposed a multi-constrained multicast routing protocol based on a genetic algorithm which determines near-optimal multicast routes on demand. Based on the multi-constrained genetic algorithm approach, the proposed routing protocol can optimize multiple QoS parameters simultaneously.

#### 5.2.4. Particle Swarm Optimization-Based Methods

The particle swarm optimization is a kind of algorithm based on swarm intelligence which was proposed by Konnedy and Eberhart in 1995 [20]. The particle swarm optimization is inspired by the foraging behavior of birds. In particle swarm optimization, every optimization solution is a bird in search space, which is called a particle. Every particle has a fitness value decided by the optimal function. The speed vector decides the direction and the distance of the particle. Then the other particles will follow the optimal particle to search for an optimal solution in the solution space. The initialization of the particle swarm optimization is a set of random solutions; then the algorithm will find the optimal solution by iteration. In each round of iteration, the particle updates itself by following two extrema. The first extremum is the optimal solution which is found by a particle, called private best. Another extremum is the present optimal solution for the whole swarm, which is the global best. In recent years, the PSO algorithm has been widely used in function optimization, neural network training, fuzzy logic control, or any research area in which genetic algorithms can be used.

The authors in [88] use a two tier particle swarm algorithm to configure a network for achieving an optimal energy consumption and packet delivery rate performance. A hybrid technique is proposed in [89], which integrates both the PSO algorithm and the GA algorithm together. In this algorithm, the constraint HC (Hybridization Cofficient) is used to express the population percentage. When HC is zero, it means the procedure is a PSO algorithm; when HC is 1, it means the procedure is a GA algorithm. A PSO-based routing and clustering protocol has been proposed in [90]. In this algorithm, the gateway collects information from nodes and sends the information to a base station. Once the base station receives the information from the gateways, it will execute PSO-based routing and clustering algorithms to form clusters. In [91], the PSO algorithm will be used to judge different paths and choose the best optimized path according to the fitness value. The fitness value represents the energy consumption in this algorithm. The endocrine cooperative particle swarm optimization (ECPSOA)-based routing recovery method is proposed in [92]. In ECPSOA, the multi-swarm strategy guides particles flying in better directions, and the endocrine mechanism yields a high diversity of particles to increase search space, which can jump out of the local optimization and improve the searching capability.

#### 5.2.5. Neural Network-Based Methods

The artificial neural network (NN) concept, proposed in the 1940s [24], is constituted by a large amount of neurons that have tunable weights. The NN has some advantages such as large scale parallel processing, distributed storage, self-organization, self-learning, *etc.* The function and structure of signal neurons are simple, but the system that consists of a large amount of neurons is complex. The NN can represent the basic characteristics of human brain. Compared with a digital computer, the NN is much closer to the human brain structure and function. The NN does not compute step by step based on specific program; it can adapt to a changing environment and summarize the rules to achieve specific functions. The first step of a NN is learning, which is based on criteria. One such criterion is that if the network makes a wrong decision, then the probability that it will make the same mistake in the future should be reduced. Therefore, in general, the more neurons in the network, the better the performance is.

In [93], each node uses the neural network to manage the routing to a sink node. The nodes use the latency, the throughput, the error rate, and the duty cycle to choose the best next hop relay node. Based on link state routing protocols and a neural network, in [94], the nodes use the number of hops, the bandwidth, the load, and the propagation delay to create metrics. The metrics use a neural network to optimize routing selection and network resource occupancy. As in a mobile *ad hoc* network, multipath routing can improve the network reliability greatly. In [95], each node in the network will be equipped with a neural network and can be trained and used to select optimal and sub-optimal high reliable disjoint paths, which can improve the network reliability remarkably.

#### 5.2.6. Reinforcement Learning-Based Methods

Reinforcement learning (RL) was proposed by Kaelbling *et al.* in 1996 [21], and Sutton and Barto in 1998 [22], respectively. RL is a sub-area of machine learning. RL uses a computer program to generate patterns or rules from a large data set. RL is inspired by animal learning. In reinforcement learning, if the behavior strategy has a positive effect on the environment, then the agent will reinforce this behavior strategy in future. The object is to find the optimization strategy in every discrete state and summarize the maximum reward. RL regards learning as a process of exploration. The agent chooses an action to act on the environment, and the environment changes according to the action. Meanwhile, the environment will generate a reinforcement signal for the agent. The agent chooses the next step action according to the environment and the reinforcement signal. The principle of action selection is to maximize the positive reward probability. The action can not only affect the value of the reinforcement signal, but also the environment status. In RL, the reinforcement signal is just an evaluation of the agent action. Due to the fact the environment can only provide a little information, the agent must be able to rely on its own experience. Based on this method, the agent can learn knowledge from the action, and modify the action strategy to adapt to the environment.

In [96], based on the machine learning concept, the authors proposed an energy efficient and lifetime extending routing protocol. In [96], the nodes calculate the Q-values of their neighbors and choose the neighbors with highest Q-value as the next hop forwarders. By making use of the RL technique, this protocol can effectively adapt the dynamic network topology. Like [96], in [97], the nodes choose the next hop relay nodes based on the node ID and the Q-value. The neighbors whose ID is the same with source node and the Q-value is highest among the neighbor nodes will be chosen as the relay nodes. The work in [98] uses reinforcement learning to provide a proactive link failure resilient routing protocol for MANETs (Mobile *Ad-Hoc* Networks). In [99], nodes in a cooperative node group will be considered as opponents to each other; therefore, each node will maintain a Q-value which reflects the payoff that would have been received if that node selects one action and the other nodes jointly selected the other action. After that, the node with the highest total payoff will be elected to forward the data packet to the next cooperative node group towards the sink node. More reinforcement learning-based routing protocols can be found in [100,101].

#### 5.2.7. Ant Colony Optimization-Based Methods

The ant colony algorithm was proposed by Colorni and Dorigo in the 1990s [19]. This algorithm is inspired by the behavior of ant colonies. The researchers found that ants communicate with each other through a kind of chemical called a pheromone. The ants can leave pheromones in the path that they pass by and perceive the pheromone during their movements. The pheromone can guide the movement of ants, so the collective behaviors of ant colony algorithms consist of a large amount of ants. The phenomenon can provide positive feedback to the algorithm to enhance the routing selection process. Therefore, once there is a route which many ants pass by, then the probability that this route will be chosen by other ants will be high. The ants can use this kind of information exchange to find food. Based on this method, the ant colony algorithm can achieve global optimization. The ant colony algorithm can be used to address combinatorial optimization problems such as the asymmetric traveling salesman problem, vehicle routing, WSN routing, *etc.*

There are many researches that use the ant colony algorithms to improve the routing performance of wireless sensor networks, such as in [102,103,104,105,106]. In [102], the ant colony algorithm works with LEACH (Low Energy Adaptive Clustering Hierarchy) to choose the best next hop relay node. In [103], the AFSA (Artificial fish swarm algorithm) algorithm finds a candidate optimal routing path roughly and the ACOA (ant colony optimization algorithm) algorithm helps the AFSA algorithm to calibrate this routing path. Similar algorithms can be found in [104,105,106]; in these algorithms the ant colony algorithms are used independently or combined with other intelligent algorithms to select the best routing or the best cluster head of the network.

#### 5.2.8. Summary

In this subsection, we introduced seven different kinds of intelligent algorithm-based routing protocols. Due to the intelligence of the intelligent algorithm, these routing protocols are more effective and robust than traditional routing protocols; moreover, they have capability to deal with the multiple constraints of a network. The learning-based intelligent algorithms such as the FL, the NN, the RL, and the ant colony have good performance in routing discovery and routing building; the SA, the GA, and the PSO are similar intelligent algorithms which are mainly used to form clusters or choose the cluster head for the network.

Unfortunately, excepting the fuzzy logic algorithm, the other six intelligent algorithms have not been used yet in the underwater environment. Although the availability and the performance of these intelligent algorithms have been talked about in terrestrial environments, considering the differences between the terrestrial environment and the underwater environment, the performance of these intelligent algorithms in the underwater environment also deserves to be investigated. The applications and research on intelligent algorithms in underwater acoustic sensor networks are just beginning; many excellent intelligent algorithms have not been implemented yet in underwater environments. Therefore, this is a highly potential research area for underwater acoustic sensor network routing protocol design, and we anticipate many open issues and challenges.

## 6. Open Issues and Challenges

Even through terrestrial wireless sensor networks have been studied for a long time and used in a wide range of applications, considering the differences between the terrestrial environment and the underwater environment, terrestrial wireless sensor network solutions cannot be directly used in underwater environments. The main challenges for the underwater acoustic sensor network routing protocol design are as follows.

- The attenuation and absorption of acoustic wave in underwater environment is more serious than those of RF waves in terrestrial environments, which means that more transmission energy will be needed in an underwater environment, especially considering that the underwater sensor nodes are energy limited.
- The propagation delay, the bandwidth, the link quality, and the bit error rate in underwater acoustic channels are worse than those of terrestrial wireless channels.
- Due to the node movement and failure, the topology of underwater acoustic sensor networks changes frequently. Moreover, the architecture of underwater acoustic sensor networks is 3-dimensional, which is different from terrestrial wireless sensor networks.
- Since underwater GPS devices and underwater location algorithms are expensive and complex, the location information of the underwater sensor nodes is hard to get.
- The devices used in underwater acoustic sensor networks are much more complex and expensive than in terrestrial WSNs, because the devices used in underwater environments need to consider the waterproofness and the corrosion resistance under water.

Since there are so many research challenges in routing protocol design for underwater acoustic sensor networks, there are also a large number of open issues that need to be investigated:

1. The propagation delay model. Due to the fact the propagation delay in underwater acoustic sensor networks is serious, how to calculate the propagation delay and build propagation models is still an unresolved issue.
2. The energy consumption model. The energy in underwater sensor nodes is strictly limited, therefore how to reduce the energy consumption of underwater acoustic sensor networks is always the most important research area. However, there is currently no accurate and reliable energy consumption model for underwater sensor nodes and networks.
3. The movement model. Due to the water currents, underwater sensor nodes move continuously. Even though there are already a lot of mobility models for terrestrial sensor nodes, the special network structure and hydromechanics make the movement of underwater sensor nodes totally different from that of terrestrial sensor nodes. An appropriate motion model is needed for underwater sensor networks.
4. High efficiency and reliable communication. In underwater acoustic channels, the bandwidth, the link quality, and the bit error rate are all worse than those of terrestrial RF channels, so how to improve the efficiency and the reliability of underwater communication channels also deserves to be investigated.
5. The utilization of intelligent algorithms in the underwater environment. This is a new research area for underwater acoustic sensor work. Since there are only a few intelligent algorithms that have been used in UASN routing protocols, therefore, how to use the intelligent algorithms to solve the issues that exist in underwater acoustic sensor networks has been a hot open issue in recent years.
6. The location information acquisition. In underwater acoustic sensor networks, the location information is useful in routing discovery, however, the necessary GPS devices and location algorithms are expensive and complex. Therefore, how to get the location information of underwater sensor nodes easily and effectively is an important open issue.

**Figure 6:**
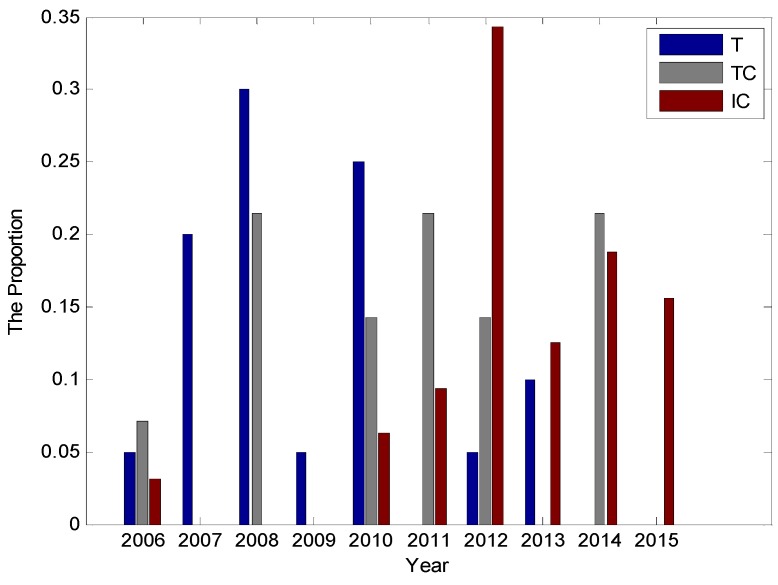
The development trends of UASN routing protocols.

To provide researchers with clear and direct insights for the development of underwater acoustic sensor network routing protocols, we show the development trends of UASN routing protocols in recent years, which can be found in Figure 6.

In Figure 6, the “T” means the traditional non-cross-layer design routing protocol, the “TC” means the traditional cross-layer design routing protocol, and the “IC” means the intelligent algorithm-based routing protocol. From Figure 6, we can conclude the following development trends in UASN routing protocols: the traditional non-cross-layer design routing protocols were mainly used before 2010, while the traditional cross-layer design routing protocols developed from 2010 to 2014, and the intelligent algorithm-based routing protocols are being utilized since 2010. Figure 6 can give researchers a clear and direct viewpoint that in the future the most hot research areas in the field of underwater acoustic sensor network routing protocol design will be the cross-layer design methods and the intelligent algorithm-based methods.

## Figures and Tables

**Table 1:** The differences between the underwater environment and the terrestrial environment.

	Underwater Environment (Acoustic Wave)	Terrestrial Environment (RF Wave)
Propagation speed	Low (1200 m/s to 1400 m/s)	High (3 × 10^8^ m/s)
Energy consumption	High	Low
Propagation delay	High	Low
Bandwidth	Low	High
Data rate	Low	High
Noise and interference	High	Low
Dynamics	High	Low
Reliability	Low	High

## Appendix

**Table A1:** The classification and the comparison of the UASN routing protocols.

			Protocol	Year	Underwater or Terrestrial	Hop-by-Hop or End-to-End	Requirements or Assumptions	Cluster or Single Entity	Hello or Control Message	Advantages
Non-Cross-Layer design protocol	Energy Efficient		DBR	2008	Underwater	Hop-by-Hop	depth information	single entity	Yes	Use the depth information instead of the location information; reduce the redundant packets transmission, energy consumption, and collision.
			DDD	2007	Underwater	single hop	AUVs needed	n/a	Yes	The communication only occurs in one-hop range, which minimal the energy consumption for the whole network.
			EUROP	2008	Underwater	hop-by-hop	depth information	single entity	Yes	Reduce the energy consumption and minimize the effect of extreme long propagation delay.
			HH-VBF	2007	Underwater	hop-by-hop	location information	single entity	No	Improving the robustness of packet delivery in sparse networks and enhancing the data delivery ration while taxing less energy than VBF.
			NIR	2010	Underwater	hop-by-hop	location information	single entity	Yes	Low level energy consumption and high probability of packet delivery.
			Mobicast	2013	Underwater	hop-by-hop	AUVs needed	clustered	No	Improving the successful delivery rate, reducing the power consumption and message overhead.
			DBMR	2010	Underwater	end-to-end	depth information	single entity	Yes	Using the multi-hop transmission model to replace the flooding model, which can make the DBMR is much more energy efficient than DBR.
			EERS	2008	Underwater	hop-by-hop	geographic information	single entity	Yes	High energy efficiency which close to the optimal energy performance, good trade-off between the throughput and delay.
			AURP	2012	Underwater	hop-by-hop	AUVs needed	single entity	Yes	The total data transmissions are minimized and the short range high data rate achieve by AUVs, high delivery ratio and low energy consumption.
	Mobility		HydroCast	2010	Underwater	hop-by-hop	pressure information	single entity	Yes	Maximizes greedy progress and limiting co-channel interference.
			DFR	2008	Underwater	hop-by-hop	location information	single entity	No	Increasing the probability of successful delivery and delivery ratio, addressing the void problem.
			VBF	2006	Underwater	end-to-end	location information	single entity	No	Scalable, robustness, and energy efficient for the highly dynamic network.
			TCBR	2010	Underwater	hop-by-hop	special mechanical module	clustered	Yes	Increasing the reliability, reducing the energy consumption, and manage the problems of node mobility.
			REBAR	2008	Underwater	hop-by-hop	location information	single entity	No	Increasing the delivery ratio and reducing the energy consumption of the nodes near the sink node.
			VAPR	2013	Underwater	hop-by-hop	depth information	single entity	Yes	Robustness to dynamic topology, can avoid the void in routing discovery.
	Time delay		UW-HSN	2008	Underwater	hop-by-hop	special mechanical module	single entity	Yes	Increase overall network capacity, lower the delays.
			H2-DAB	2009	Underwater	hop-by-hop	n/a	single entity	Yes	Minimize the message latency; reduce the energy consumption without any extra or specialized network equipment.
			ICRP	2007	Underwater	end-to-end	n/a	single entity	No	Combine the routing discovery and the data transmission together; improve the energy efficient, scalable, and the reliability of the data paths.
			DUCS	2007	Underwater	hop-by-hop	n/a	clustered	Yes	Minimizes the proactive routing exchange, can adapt the node mobility, reduce the interference and improve the communication quality.
			MPR	2010	Underwater	hop-by-hop	n/a	single entity	Yes	Low propagation delay, adaptive to the node mobility, can achieve load balance.
Cross-Layer design protocol	Traditional cross-layer design routing protocol	Location information free	CARP	2014	Underwater	hop-by-hop	history of the successful transmission	single entity	Yes	Use the history of the successful transmission to select the next hop, improving the robustness and deliver ratio, reducing the energy consumption.
			UMIMO	2012	Underwater	hop-by-hop	special mechanical module	single entity	Yes	Leverage the tradeoff between multiplexing and diversity gain, select suitable subcarriers to avoid interference.
			E-PULRP	2010	Underwater	hop-by-hop	pre-defined layer	clustered	Yes	Reducing the energy consumption, can adaptive the mobility of the network, prolong the network lifetime.
			ERP2R	2011	Underwater	hop-by-hop	n/a	single entity	Yes	Balance the energy consumption, prolong the network lifetime, and reduce the end-to-end delay and energy consumption.
			APCR	2012	Underwater	hop-by-hop	n/a	clustered	Yes	Achieve high delivery ratio and low energy consumption, reducing the delay in both sparse and dense networks.
			EEIA	2014	Underwater	hop-by-hop	n/a	single entity	Yes	Propose a set of routing protocol which can reduce the energy consumption and the interference.
			EEDBR	2011	Underwater	hop-by-hop	depth information	single entity	Yes	Set different holding time according the residual energy, reduce the energy consumption and prolong the network lifetime.
			TBRD	2011	Underwater	end-to-end	special mechanical module	clustered	Yes	Reducing the energy consumption, the end-to-end delay, and the probability of the packet dropping.
			EADA-RAT	2008	Underwater	end-to-end	sensor ID	single entity	Yes	Energy saving by minimizing the number of data transmissions, decreases the delay by automatic movement of the aggregation point, and extends the network lifetime.
		Location information needed	AHH-VBF	2014	Underwater	hop-by-hop	location information	single entity	No	Improving the data delivery ration, energy consumption, and end-to-end latency compared to the HH-VBF.
			FBR	2008	Underwater	hop-by-hop	location information	single entity	Yes	Reduce the energy per bit consumption and average packet end-to-end delay.
			SEANAR	2010	Underwater	hop-by-hop	location information	single entity	Yes	Assign bigger weight to node with high connectivity to the sink, which increase the packet delivery ratio, and keep the energy consumption in a low level.
			DIDS	2006	Underwater	hop-by-hop	location information	single entity	Yes	Minimizing the energy consumption, consider the underwater channel and the application requirement.
			ARDDT	2008	Underwater	hop-by-hop	location information	single entity	Yes	Satisfy different application requirement, achieve a good trade-off among delivery ratio, average end-to-end delay, and energy consumption.
	Intelligent algorithm based routing protocol	FL	PER	2011	Underwater	hop-by-hop	n/a	single entity	Yes	Can achieve excellent performance in terms of the metrics, the packet delivery ratio, energy consumption and average end-to-end delay.
			CBRA	2014	Underwater	single hop	location information	clustered	Yes	Reducing the energy consumption and prolong the network lifetime by using the fuzzy logic system.
			DREE	2015	Underwater	hop-by-hop	n/a	single entity	Yes	The protocol outperforms network lifetime, energy consumption, and data delivery ration by utilizing the fuzzy logic based link estimator.
			GBFO	2015	Underwater	hop-by-hop	n/a	clustered	No	Reducing the energy consumption and end-to-end delay, prolong the network lifetime.
			FBCA	2014	Underwater	single hop	location information	clustered	Yes	High throughput, delivery ratio; low delay and energy consumption.
		SA	LEACH-C	2002	Terrestrial	single hop	location information	clustered	Yes	Self-organization, save communication resources, improves the system lifetime.
			EELEACH-C	2012	Terrestrial	single hop	n/a	clustered	Yes	Minish the total energy consumption, prolong the network lifetime.
			EERS	2012	Terrestrial	single hop	location information	clustered	Yes	Global optimization, cost effective, improve the routing success ratio and reduce the routing cost.
			LER	2012	Terrestrial	end-to-end	n/a	single entity	No	Can deal with the mobility of the sink, higher efficiency in terms of the packet transmission distance, the hop counts, and the energy consumption.
			ILEACH	2013	Terrestrial	single hop	location information	clustered	Yes	The performance of the energy consumption and network lifetime has been improved by introducing the VCH to the algorithm, which can reduce the frequency re-clustering.
		GA	GAOUP	2011	Terrestrial	end-to-end	location information	single entity	Yes	Development time is much shorter than the traditional approaches; the systems are robust and insensitive to noisy and missing data.
			ERP	2012	Terrestrial	single hop	n/a	clustered	No	New fitness function is proposed, prolong the network lifetime and stability period, and reduce the energy consumption.
			ORGA	2012	Terrestrial	end-to-end	n/a	single entity	No	Solving the shortest path problem by using GA algorithm, and performs better and effectively when the node mobility or the topology changes.
			FMQM	2011	Terrestrial	end-to-end	n/a	single entity	No	Decrease the search space, simplify the process of coding and decoding, reduces the energy consumption.
		PSO	TPSO-CR	2015	Terrestrial	single hop	n/a	clustered	Yes	Improve the packet delivery rate at both the cluster heads and the base station, increase network coverage and maintain acceptable energy consumption at the same time.
			PSOR	2012	Terrestrial	end-to-end	n/a	single entity	No	Energy efficiency and the path to the destination node are optimized.
			PSO-GA	2014	Terrestrial	end-to-end	n/a	single entity	No	Higher precision and lower computational complexity, the performance is better than PSO and GA.
			EECR	2014	Terrestrial	single hop	n/a	clustered	Yes	The network lifetime, the number of inactive sensor nodes, and the total data packets transmission are better than the existing algorithms.
			ECPSOA	2015	Terrestrial	end-to-end	location information	single entity	Yes	Reduce the communication overhead in terms of both energy and delay, and the network robustness against path breakage due to multiple sinks movement or nodes failure is also improved.
		RL	QELAR	2010	Terrestrial	hop-by-hop	n/a	single entity	Yes	Learn the environment effectively to better adapt the dynamic networks, reduce networking overhead for higher energy efficiency, and make the energy consumption more evenly.
			FQR	2012	Terrestrial	hop-by-hop	location information	single entity	Yes	Increase the application level throughput and the link failure resiliency, and balance the energy consumption.
			EIER	2015	Terrestrial	hop-by-hop	n/a	clustered	Yes	Increase the network lifetime, the packet delivery ratio, and the network balance; reduce the packet delay.
			RECC	2013	Terrestrial	hop-by-hop	n/a	single entity	No	Efficient in terms of percentage of lost packets, network energy consumption, maximal energy consumption per node, and network lifetime.
			EQR-RL	2014	Terrestrial	hop-by-hop	n/a	single entity	Yes	Enhance the performance of the network lifetime, the end-to-end delay, and the packet delivery ration.
			DTRB	2013	Terrestrial	hop-by-hop	n/a	single entity	Yes	Deliver more messages than a traditional delay tolerant one in densely populated areas.
		NN	SIR	2006	Terrestrial	end-to-end	n/a	clustered	No	Can achieve superior performance in terms of average latency and energy consumption over the traditional routing, and prolong the network lifetime.
			NNBH	2012	Terrestrial	hop-by-hop	n/a	single entity	Yes	Scalable and adapt for dynamic network topology and real network environment.
			TCNN	2012	Terrestrial	end-to-end	n/a	single entity	Yes	The disjoint path set reliability is much higher than the shortest one, the reliability and the number of paths are improve, and the number of paths in the path the set is also improved.
		ACO	LEACH-P	2012	Terrestrial	end-to-end	n/a	clustered	Yes	Prolong the network lifetime, balance the energy consumption.
			ACOA-AFSA	2012	Underwater	end-to-end	n/a	single entity	No	Have better performance on energy consumption, packet loss rate, and time delay than VBF and LEACH.
			EAAR	2010	Terrestrial	end-to-end	n/a	single entity	Yes	Reducing the energy consumption of the nodes and prolong the network lifetime.
			FACOR	2014	Terrestrial	end-to-end	n/a	single entity	Yes	Increasing the network lifetime, reducing the energy consumption, and increasing the packet delivery ratio.
			AOCR	2013	Terrestrial	end-to-end	n/a	clustered	Yes	Achieve better results in terms of packets delivery time and residual network energy.

## References

[1] Benson B., Li Y., Kastner R., Faunce B., Domond K., Kimball D., Schurgers C. Design of a Low-Cost Underwater Acoustic Modem for Short-Range Sensor Networking Applications. Proceeding of the 2010 IEEE OCEANS.

[2] Heidemann J., Stojanovic M., Zorzi M. (2012). Underwater sensor networks: Applications, advances and challenges. Philos. Trans. R. Soc..

[3] Ayaz M., Baig I., Abdullah A., Faye I. (2011). A survey on routing techniques in underwater wireless sensor networks. J. Netw. Comput. Appl..

[4] Akyildiz I.F., Pompili D., Melodia T. State-of-the-Art in Protocol Research for Underwater Acoustic Sensor Networks. Proceeding of the 1st ACM International Workshop on Underwater Networks.

[5] Climent S., Sanchez A., Capella J.V., Meratnia N., Serrano J.J. (2014). Underwater Acoustic Wireless Sensor Networks: Advances and Future Trends in Physical, MAC and Routing Layers. Sensors.

[6] Tanebbaum A.S. (2011). Computer Networks.

[7] Wu J. (2005). Handbook on Theoretical and Algorithmic Aspects of Sensor, Ad Hoc Wireless, and Peer-to-Peer Networks.

[8] Lu X.L. (2008). The Research on *Ad Hoc* Network Cross-Layer Design. Ph.D. Thesis.

[9] Chiang M., Low S.H., Calderbank R.A., Doyle J.C. (2007). Layering as Optimization Decomposition: A Mathematical Theory of Network Architectures. Proc. IEEE.

[10] Elbatt T., Ephremides A. (2004). Joint Scheduling and Power Control for Wireless *Ad-Hoc* Networks. IEEE Trans. Wirel. Commun..

[11] Radunovic B., Boudec J.Y.L. Joint scheduling, power control and routing in symmetric, one dimensional, multi-hop wireless networks. Proceedings of the Modeling and Optimization in Mobile, *Ad-Hoc* and Wireless Networks.

[12] Tsatsanis M.K., Zhang R., Banerjee S. (2000). Network assisted diversity for random access wireless networks. IEEE Trans. Signal Process..

[13] So J., Vaidya N. Multi-channel MAC for *Ad-Hoc* networks: Handling multi-channel hidden terminals using a single transceiver. Proceeding of the 5th ACM International Symposium on Mobile *Ad-Hoc* Networking and Computing.

[14] Cao M., Raghunathan V., Kumar P.R. Cross-Layer Exploitation of MAC Layer Diversity in Wireless Networks. Proceedings of the 14th IEEE International Conference on Network Protocols.

[15] Xu Y., Heidemann J., Estrin D. (2000). Adaptive Energy Conserving Routing for Multihop Ad Hoc Networks.

[16] Setton E., Yoo T., Zhu X.Q. (2005). Cross-Layer Design of *Ad-Hoc* Networks for Real time Video Streaming. IEEE Wirel. Commun..

[17] Metropolis N., Rosenbluth A.W., Rosenbluth M.N., Teller A.H., Teller E. (1953). Equation of State Calculations by Fast Computing Machines. J. Chem. Phys..

[18] Holland J.H. (1975). Adaptation in Natural and Artificial Systems: An Introductory Analysis with Applications to Biology, Control, and Artificial Intelligence.

[19] Dorigo M., Gambardella L.M. (1997). Ant colony system: A cooperative learning approach to the traveling salesman problem. IEEE Trans. Evolut. Comput..

[20] Kennedy J., Eberhart R. Particle swarm optimization. Proceedings of the IEEE International Conference on Neural Networks.

[21] Kaelbling L.P., Littman M.L., Moore A.W. (1996). Reinforcement Learning: A Survey. J. Artif. Intell. Res..

[22] Sutton R.S., Barto A.G. (1998). Reinforcement Learning: An Introduction.

[23] Zadeh L.A. (1965). Fuzzy sets. Inf. Control.

[24] McCulloch W.S., Pitts W. (1943). A logical calculus of the ideas immanent in nervous activity. Bull. Math. Biophys..

[25] Kirkpatrick S., Gelatt C.D., Vecchi M.P. (1983). Optimization by Simulated Annealing. Science.

[26] Chen K.Y., Ma M.D., Cheng E., Yuan F., Su W. (2014). A Survey on MAC Protocols for Underwater Wireless Sensor Networks. IEEE Commun. Surv. Tutor..

[27] Kredo K.B., Mohapatra P. A Hybrid Medium Access Control Protocol for Underwater Wireless Networks. Proceedings of the Thirteenth Annual International Conference on Mobile Computing and Networking.

[28] Casari P., Zorzi M. (2011). Protocol Design Issues in Underwater Acoustic Networks. Comput. Commun..

[29] Wang Q., Hempstead M., Yang W. A Realistic Power Consumption Model for Wireless Sensor Network Devices. Proceedings of the 3rd Annual IEEE Communications Society on Sensor and *Ad-Hoc* Communications and Networks.

[30] Dunkels A., Osterlind F., Tsifites N., He Z. Software-Based On-Line Energy Estimation for Sensor Nodes. Proceedings of the 4th Workshop on Embedded Networked Sensors.

[31] Kellner S., Pink M., Meier D., Blass E. Towards a Realistic Energy Model for Wireless Sensor Networks. Proceedings of the 5th Annual Conference on Wireless on Demand Networks Systems and Services.

[32] Wang Q., Yang W. Energy Consumption Model for Power Management in Wireless Sensor Networks. Proceedings of the 4th Annual IEEE Communications Society Conference on Sensor, Mesh and *Ad-Hoc* Communications and Networks.

[33] Shareef A., Zhu Y. Energy Modeling of Wireless Sensor Nodes Based on Petri Nets. Proceedings of the 39th International Conference on Parallel Processing.

[34] Zhou H., Luo D., Gao Y., Zuo D. (2011). Modeling of Node Energy Consumption for Wireless Sensor Networks. Wirel. Sens. Netw..

[35] Domingo M.C., Prior R. (2008). Energy analysis of routing protocols for underwater wireless sensor networks. Comput. Commun..

[36] Bettstetter C. (2004). Mobility Modeling, Connectivity, and Adaptive Clustering in *Ad-Hoc* Networks. Ph.D. Thesis.

[37] Zhou Z., Peng Z., Cui J.H., Shi Z.J. (2010). Scalable Localization with Mobility Prediction for Underwater Sensor Networks. IEEE Trans. Mob. Comput..

[38] Erol M., Vieira L.F.M., Gerla M. Localization with Dive’N’Rise (DNR) beacons for underwater acoustic sensor networks. Proceeding of the Second Workshop on Underwater Networks.

[39] Caruso A., Paparella F., Vieira L.F.M., Erol M. The Meandering Current Mobility Model and its Impact on Underwater Mobile Sensor Networks. Proceeding of the 27th IEEE Conference on Computer Communications.

[40] Yan H., Shi Z.J., Cui J.H. (2008). DBR: Depth-Based Routing for Underwater Sensor Networks. NETWORKING 2008 Ad Hoc and Sensor Networks, Wireless Networks, Next Generation Internet.

[41] Magistretti E., Kong J.J., Lee U., Gerla M., Bellavista P., Corradi A. A Mobile Delay-Tolerant Approach to Long-Term Energy-Efficient Underwater Sensor Networking. Proceeding of the IEEE Wireless Communications and Networking Conference.

[42] Yang C.H., Ssu K.F. An energy-efficient routing protocol in underwater sensor networks. Proceeding of the 3rd International Conference on Sending Technology.

[43] Jiang Y.F., Lin S. NIR: UWSN Routing Protocol Based on Node Neighbor Information. Proceeding of the International Conference on Future Information Technology and Management Engineering.

[44] Chen Y.S., Lin Y.W. (2012). Mobicast Routing Protocol for Underwater Sensor Networks. IEEE Sens. J..

[45] Liu G.Z., Li Z.B. Depth-Based Multi-Hop Routing Protocol for Underwater Sensor Network. Proceeding of the 2nd International Conference on Industrial Mechatronics and Automation.

[46] Zorzi M., Casari P., Baldo N., Harris A.F. (2008). Energy-Efficient Routing Schemes for Underwater Acoustic Networks. IEEE Sel. Areas Commun..

[47] Yoon S., Azad A.K., Hoon O., Kim S.H. (2012). AURP: An AUV-Aided Underwater Routing Protocol for Underwater Acoustic Sensor Networks. Sensors.

[48] Ayaz M., Abdullah A., Jung L.T. Temporary cluster based routing for Underwater Wireless Sensor Networks. Proceedings of the International Symposium in Information Technology.

[49] Domingo M.C., Prior R. A Distributed Clustering Scheme for Underwater Wireless Sensor Networks. Proceedings of the 18th International Personal, Indoor and Mobile Radio Communications.

[50] Lee U., Wang P., Noh Y., Vieira F.L.M. Pressure Routing for Underwater Sensor Networks. Proceedings of the 2010 IEEE INFOCOM.

[51] Hwang D., Kim D. DFR: Directional flooding-based routing protocol for underwater sensor networks. Proceedings of the OCEANS 2008.

[52] Xie P., Cui J.H., Li L. VBF: Vector-Based Forwarding Protocol for Underwater Sensor Networks. Proceedings of the 5th International IFIP-TC6 Networking Conference.

[53] Nicolaou N., See A., Xie P., Cui J.H. Improving the Robustness of Location-Based Routing for Underwater Sensor Networks. Proceeding of the IEEE OCEANS Europe Conference.

[54] Chen J.M., Wu X.B., Chen G.H. REBAR: A Reliable and Energy Balanced Routing for UWSNs. Proceedings of the 7th International Conference on Grid and Cooperative Computing.

[55] Youngtae N., Uichin L., Paul W., Choi B.S.C. (2012). VARP: Void-Aware Pressure Routing for Underwater Sensor Networks. IEEE Trans. Mob. Comput..

[56] Ali K., Hassanein H. Underwater Wireless Hybrid Sensor Networks. Proceedings of IEEE Symposium on Computers and Communications.

[57] Ayaz M., Abdullah A. Hop-by-Hop Dynamic Addressing Based (H2-DAB) Routing Protocol for Underwater Wireless Sensor Networks. Proceedings of the International Conference on Information and Multimedia Technology.

[58] Liang W., Yu H.B., Liu L., Li B.X. Information-Carrying Based Routing Protocol for Underwater Acoustic Sensor Networks. Proceedings of the International Conference on Mechatronics and Automation.

[59] Chen Y.S., Juang T.Y., Lin Y.W., Tsai I.C. (2010). A Low Propagation Delay Multi-Path Protocol for Underwater Sensor Networks. J. Internet Technol..

[60] Basagni S., Petrioli C., Petroccia R., Spaccini D. Channel-aware routing for underwater wireless networks. Proceedings of the OCEANS.

[61] Kuo L.C., Melodia T. Cross-Layer routing on MIMO-OFDM underwater acoustic links. Proceedings of the 9th Annual IEEE Communications Society Conference on Sensor, Mesh and *Ad-Hoc* Communications and Networks.

[62] Gopi S., Kannan G., Desai U.B., Merchant S.N. Energy Optimized Path Unaware Layered Routing Protocol for Underwater Sensor Networks. Proceedings of the IEEE Global Telecommunications Conference.

[63] Wahid A., Lee S., Kim D. An energy-efficient routing protocol for UWSNs using physical distance and residual energy. Proceedings of the OCEANS.

[64] Shasshaj A., Petroccia R., Petrioli C. Energy efficient interference-aware routing and scheduling in underwater sensor networks. Proceedings of the OCEANS.

[65] Bzoor M.A., Zhu Y.B., Liu J., Reda A., Cui J.H., Rajasekaran S. (2012). Adaptive Power Controlled Routing for Underwater Sensor Networks. Wirel. Algorithms Syst. Appl..

[66] Wahid A., Lee S., Jeong H.J., Kim D. (2011). EEDBR: Energy-Efficient Depth-Based Routing for Underwater Wireless Sensor Networks. Adv. Comput. Sci. Inf. Technol..

[67] Kuo L.C., Melodia T. Tier-Based Underwater Acoustic Routing for Applications with Reliability and Delay Constraints. Proceedings of the IEEE International Workshop on Wireless Mesh and *Ad-Hoc* Networks.

[68] Nam H., An S. Energy-Efficient Routing Protocol in Underwater Acoustic Sensor Networks. Proceedings of the IEEE/IFIP International Conference on Embedded and Ubiquitous Computing.

[69] Yu H., Yao N., Liu J. (2015). An adaptive routing protocol in underwater sparse acoustic sensor networks. Ad Hoc Netw..

[70] Jornet J.M., Stojanovic M., Zorzi M. Focused beam routing protocol for underwater networks. Proceedings of the Third ACM International workshop on Underwater Networks.

[71] Wu X., Chen G., Chen J. Energy-Efficient and Topology-Aware Routing for Underwater Sensor Networks. Proceedings of the 19th International Conference on Computer Communications and Networks.

[72] Pompili D., Melodia T., Akyildiz I.F. Routing algorithm for delay-insensitive and delay-sensitive applications in underwater sensor networks. Proceedings of the 12th Annual International Conference on Mobile Computing and Networking.

[73] Guo Z., Colombo G., Wang B., Cui J.H. Adaptive Routing in Underwater Delay/Disruption Tolerant Sensor Networks. Proceedings of the Fifth Annual Conference on Wireless on Demand Network Systems and Services.

[74] Huang C.J., Wang Y.W., Liao H.H., Lin C.F., Hu K.W., Chang T.Y. (2011). A power-efficient routing protocol for underwater wireless sensor networks. Appl. Soft Comput..

[75] Banerjee R., Bhattacharyya C.K. Cluster based routing algorithm with evenly load distribution for large scale networks. Proceedings of the International Conference on Computer Communication and Informatics.

[76] Tariq M., Latiff M.S., Ayaz M., Coulibaly Y., Al-Areqi N. (2015). Distance based Reliable and Energy Efficient (DREE) Routing for Underwater Acoustic Sensor Networks. J. Netw..

[77] Reza M.M., Rahman K.T., Zakaria A.S.M. (2015). Grid based Fuzzy Optimized Routing Protocol for Underwater Sensor Networks. Int. J. Comput. Appl..

[78] Goyal N., Dave M., Verma A.K. Fuzzy based clustering and aggregation technique for underwater wireless sensor networks. Proceedings of the International Conference on Electronics and Communication Systems.

[79] Heinzelman W.B., Chandrakasan A.P., Balakrishnan H. (2002). An application-specific protocol architecture for wireless microsensor networks. IEEE Trans. Wirel. Commun..

[80] Shi S., Liu X., Gu X. An energy-efficiency Optimized LEACH-C for wireless sensor networks. Proceedings of the 7th International ICST Conference on Communications and Networking in China.

[81] Kaura R., Majithia S. (2012). Efficient End to End Routing using RSSI and Simulated Annealing. Int. J. Eng. Res. Technol..

[82] Zhao F., Xu Y., Li R. (2012). Improved LEACH Routing Communication Protocol for a Wireless Sensor Network. Int. J. Distrib. Sens. Netw..

[83] Han S.W., Jeong I.S., Kang S.H. (2013). Low Latency and energy efficient routing tree for wireless sensor networks with multiple mobile sinks. J. Netw. Comput. Appl..

[84] Chakraborty A., Mitra S.K., Naskar M.K. (2011). A Genetic Algorithm inspired Routing Protocol for Wireless Sensor Networks. Int. J. Comput. Intell. Theory Pract..

[85] Attea B.A., Khalil E.A. (2012). A new evolutionary based routing protocol for clustered heterogeneous wireless sensor networks. Appl. Soft Comput..

[86] Mohammed A.A., Nagib G. (2012). Optimal Routing In *Ad-Hoc* Networking Using Genetic Algorithm. Int. J. Adv. Netw. Appl..

[87] Yen Y.S., Chao H.C., Chang R.S., Vasilakos A. (2011). Flooding-Limited and multi-constrained QoS multicast routing based on the genetic algorithm for MANETs. Math. Comput. Model..

[88] Elhabyan R.S.Y., Yagoub M.C.E. (2015). Two-tier particle swarm optimization protocol for clustering and routing in wireless sensor network. J. Netw. Comput. Appl..

[89] Lakshmanan L., Tomar D.C. (2014). Optimizing Localization Route Using Particle Swarm-A Genetic Approach. Am. J. Appl. Sci..

[90] Kuila P., Jana P.K. (2014). Energy efficient clustering and routing algorithms for wireless sensor networks: Particle swarm optimization approach. Eng. Appl. Artif. Intell..

[91] Sarangi S., Thankchan B. (2012). A Novel Routing Algorithm for Wireless Sensor Network Using Particle Swarm Optimization. Int. J. Res. Eng. Inf. Soc. Sci..

[92] Hu Y.F., Ding Y.S., Ren L.H., Hao K.R., Han H. (2015). An endocrine cooperative particle swarm optimization algorithm for routing recovery problem of wireless sensor networks with multiple mobile sinks. Inf. Sci..

[93] Barbancho J., Leon C., Molina J., Barbancho A. (2006). SIR: A New Wireless Sensor Network Routing Protocol Based on Artificial Intelligence. Adv. Web Netw. Technol. Appl..

[94] Kojic N., Reljin I., Reljin B. (2012). A Neural Network-Based Hybrid Routing Protocol for Wireless Mesh Networks. Sensors.

[95] Sheikhan M., Hemmati E. (2012). Transient chaotic neural network-based disjoint multipath routing for mobile ad-hoc networks. Neural Comput. Appl..

[96] Hu T., Fei Y. (2010). QELAR: A Machine-Learning-Based Adaptive Routing Protocol for Energy-Efficient and Lifetime-Extended Underwater Sensor Networks. IEEE Trans. Mob. Comput..

[97] Kiani F., Amir E., Zamani M., Khodadadi T., Manaf A.A. (2015). Efficient Intelligent Energy Routing Protocol in Wireless Sensor Networks. Int. J. Distrib. Sens. Netw..

[98] Oddi G., Macone D., Pietrabissa A., Liberati F. A proactive link-failure resilient routing protocol for MANETs based on reinforcement learning. Proceedings of the 20th Mediterranean Conference on Control and Automation.

[99] Maalej M., Cherif S., Basbes H. (2013). QoS and Energy Aware Cooperative Routing Protocol for Wildfire Monitoring Wireless Sensor Networks. Sci. World J..

[100] Rolla V.G., Curado M. (2013). A reinforcement learning-based routing for delay tolerant networks. Eng. Appl. Artif. Intell..

[101] Jafarzadeh S.Z., Moghaddam M.H.Y. Design of energy-aware QoS routing algorithm in wireless sensor networks using reinforcement learning. Proceedings of the 4th International Conference on Computer and Knowledge Engineering.

[102] Liao M.H., Zhang H., Sun G. (2012). Energy Aware Routing Algorithm for Wireless Sensor Network Based on Ant Colony Principle. J. Converg. Inf. Technol..

[103] Wu H.F., Chen X.Q., Shi C.J., Xiao Y.J., Xu M. (2012). An ACOA-AFSA Fusion Routing Algorithm For Underwater Wireless Sensor Network. Int. J. Distrib. Sens. Netw..

[104] Misra S., Dhurandher S.K., Obaidat M.S., Gupta P., Verma K., Narula P. (2010). An ant swarm-inspired energy-aware routing protocol for wireless *Ad-Hoc* networks. J. Syst. Softw..

[105] Amiri E., KEshavarz H., Alizadeh M., Zamani M., Khodadadi T. (2014). Energy Efficient Routing in Wireless Sensor Networks Based on Fuzzy Ant Colony Optimization. Int. J. Distrib. Sens. Netw..

[106] Li K.H., Leu J.S., Hoek J. Ant-Based On-Demand Clustering Routing Protocol for Mobile *Ad-Hoc* Networks. Proceedings of the 7th International Innovative Mobile and Internet Services in Ubiquitous Computing.

